# Human Adenovirus Type 7 Infections in Hubei, China During 2018-2019: Epidemic Features and Genetic Characterization of the Detected Viruses

**DOI:** 10.3389/fcimb.2021.684606

**Published:** 2021-08-19

**Authors:** Ying Li, Decheng Wang, Jingjing Zhang, Peiqi Huang, Hui Du, Jiali Xu, Hebin Chen, Yi Yan, Hongwei Chen, Xiaoxia Lu, Di Liu

**Affiliations:** ^1^Wuhan Children's Hospital, Tongji Medical College, Huazhong University of Science and Technology, Wuhan, China; ^2^CAS Key Laboratory of Special Pathogens and Biosafety, Wuhan Institute of Virology, Center for Biosafety Mega-Science, Chinese Academy of Sciences, Wuhan, China; ^3^National Virus Resource Center, Chinese Academy of Sciences, Wuhan Institute of Virology, Center for Biosafety Mega-Science, Wuhan, China; ^4^Wuhan Institute of Virology, University of Chinese Academy of Sciences, Beijing, China; ^5^Key Laboratory of Environment and Health (HUST), Ministry of Education & Ministry of Environmental Protection, and State Key Laboratory of Environmental Health (Incubation), School of Public Health, Tongji Medical College, Huazhong University of Science and Technology, Wuhan, China

**Keywords:** human adenovirus type 7 (HAdV-7), hemophagocytic lymphohistiocystosis (HLH), children, epidemic features, genome characterization

## Abstract

Human adenoviruses (HAdVs) type 7 can cause severe respiratory disease. During the period between December 2018 and August 2019, HAdV-7 infection was identified in 129 patients in Wuhan Children’s Hospital, Hubei Province, China. Samples were collected from hospitalized children and metagenomic sequencing was applied to detect the HAdV infections. Hemophagocytic lymphohistiocystosis (HLH) related to HAdV infections was observed in some patients clinically and patients were divided into two groups based on this to test the differences among clinical indicators. Genome variation, *in silico* restriction endonuclease analysis (REA), and phylogenetic analyses were carried out to show the genome characterization of HAdV-7 in this study. It was found that many indicators, such as all blood routine indicators, in patients of the HLH group showed significant levels. In this study, REA revealed that HAdV-7 might belong to genome 7d and genome variation analysis displayed the stable genome of HAdV. HAdV-7 is an ongoing threat to the public, and global surveillance should be established.

## Introduction

Human adenoviruses (HAdVs) are one of the most common pathogens responsible for respiratory infections. They are recognized as the cause of a range of diseases including ocular, gastrointestinal and genitourinary systems, even metabolic disorder (obesity) (Walsh et al., 2010; Lion, 2014). HAdVs belonging to the genus *Mastadenovirus* and family *Adenoviridae* are nonenveloped, double-stranded linear DNA viruses with hexon, fiber, and penton base as the three major capsid proteins (Davison et al., 2003; Cai et al., 2020). Whole genome data and bioinformatics tools have provided criteria for HAdV classification, and 104 genotypes have been assigned by the Human Adenovirus Working Group (April 2021 update) (http://hadvwg.gmu.edu/).

Since being discovered in 1953, HAdVs have been documented as the cause of local epidemics around the world (Yao et al., 2019). It has been reported that HAdVs have caused outbreaks in China, Korea, and other Asian countries (Mei et al., 2003; Chen et al., 2004; Lee et al., 2010a). From January 2010 to June 2012 the detection rate of HAdV from the examined cases of community-acquired pneumonia in children requiring hospitalization was 11% in the United States (Jain et al., 2015). HAdV-related respiratory infections were also reported in Africa with higher prevalence, such as South Africa (31.4%), Cameroon (27.3%), Senegal (50%), and Kenya (29.6%) (Breiman et al., 2015; Annamalay et al., 2016; Kenmoe et al., 2016; Assane et al., 2018). HAdV infections are mild or moderate in most cases, but it is fatal for young children, elderly or immunocompromised people when infected by HAdVs (Kandel et al., 2010; Lee et al., 2010a; Lion, 2014). Of several genotypes associated with respiratory disease, HAdV-4 and HAdV-7 are the most frequently detected and related to febrile respiratory disease of variable severity, usually leading to acute respiratory disease (ARD) (Erdman et al., 2002; Lion, 2014; Zhao et al., 2014; Yu et al., 2016). Especially HAdV-7, which have been circulating globally for decades and widely in civilian populations, and which could cause more severe respiratory illness and a higher mortality rate than other HAdVs types (Erdman et al., 2002; Fu et al., 2019). Based on the difference of restriction of endonuclease sites on the virus genome, HAdV-7 has evolved into several genome types such as 7b, 7d, 7l, and 7p (Zhao et al., 2014). Notably, the origin of HAdV-7d was recognized as China, where it circulated from 1958 to 1990 and replaced genome type HAdV-7b as the predominant epidemic strain (Wadell et al., 1985; Li et al., 1996; Zhao et al., 2014). After this it vanished for almost twenty years, and HAdV-7d re-emerged in China in 2009 and caused an outbreak in Guangdong Province in 2011 (Zhao et al., 2014).

HAdV-7 caused an outbreak in children in Hubei Province, China from December 2018 to August 2019. In this study, the prevalence, clinical data of patients, and meta-genome sequencing data are available. Hemophagocytic lymphohistiocytosis (HLH) was observed in some patients and statistics tests of clinical signs between the patient group with HLH and group without HLH were performed. Genome mutation and phylogenetic analysis were carried out to characterize the virus associated with this epidemic. By now, China has not established national surveillance for HAdV infections, meaning this epidemic in Hubei Province is a subject of concern that needs more attention. The findings in this study highlight the HLH caused by HAdV-7, the evolution of viruses, and the importance of surveillance for HAdV.

## Materials and Methods

### Patients and Specimens

The study was performed from December 1, 2018, to August 31, 2019, at Wuhan Children’s Hospital, a tertiary care hospital in Wuhan. Patients were enrolled according to the inclusion criteria: (1) patients with symptoms such as: fever, cough, wheezing; (2) imaging examination confirmed inflammation in the lungs; (3) Adenovirus can be found in bronchoalveolar lavage fluid or serum samples from patients. Patients in the Hemophagocytic lymphohistiocystosis (HLH) group met the criteria for HLH using the HScore. The medical records of each patient, including demographic data, clinical features, laboratory tests, and radiological results, were obtained. The study protocol was approved by the ethics committee of Wuhan Children’s Hospital.

### The Diagnostic Criteria of HLH

The HScore was developed by Fardet et al. and colleagues to estimate an individual’s risk of having HLH. This scoring system is freely available online (http://saintantoine.aphp.fr/score/) and contains nine items in routine practice, including known underlying immunodepression, maximal temperature (°C), hepatomegaly, splenomegaly, lower hemoglobin level, lower leucocyte count, lower platelet count, higher ferritin level (ng/ml), higher triglyceride level (mmol/l), lower fibrinogen level (g/l), higher serum glutamic oxaloacetic transaminase/recombinant aspartate aminotransferase (SGOT/ASAT) level (UI/L), hemophagocytosis features on bone marrow aspirate.

### Statistical Analysis

The independence test between HLH and co-infection was carried out in python (https://www.python.org/) with function chi2_contingency in package scipy.stats (version 1.6.3) (https://www.scipy.org/) (Virtanen et al., 2020). Clinical data were divided into two groups according to whether HLH was observed in the patient. Outliers in data were filtered according to the standard of a boxplot. Student’s t-test in python v3.6 package scipy was used to statistically analyze the difference between the mean of two group data, with statistical significance set at p-value < 0.05.

### Next-Generation Sequencing and Raw Data Processing

Blood or bronchoalveolar lavage fluid samples were handled and sequenced with PMSeq^®^ high-throughput genetic detection of pathogenic micro-organisms service provided by BGI corporation (Wuhan, Hubei Province, China). Samples were processed in a medical laboratory according to the next-generation sequencing assay manual (BGISEQ-50). The general detection process was: 1) DNA extraction from samples and quantification of nucleotide concentration; 2) cDNA libraries preparation and validation by 2100 Bioanalyzer system (Agilent Technologies, Inc.); 3). Sequencing by BGISEQ-50. Micro-organisms were detected by the bioinformatics pipeline developed by BGI (Ye et al., 2016) Clean reads after quality control and filtered by Trimmomatic were also used for genome assembly.

### Assembly of the Virus Genome

Megahit was used for de-novo assembling of clean reads to get contigs (Li et al., 2015) and the longest contigs were queried in the NCBI nucleotide database by the BLASTN program to find the most similar sequence. Reference genomes (MN307150) determined from contigs blast results were downloaded from Genbank. A mapping-based method was applied for genome assembly and clean reads were mapped to the reference genome by samtools and bcftools mpileup with all parameters set to default to obtain nearly full-length virus genome and sequencing depth (Li et al., 2009). Only 128 genomes were used for further analysis.

### Detection of Point Mutations in Virus Genome and Similarities Among Sequences

The dataset consisted of HAdV genomes in this study and the reference sequence mentioned above, and sequence alignment was performed in MAFFT (Katoh et al., 2019). BioEdit was applied to check whether there was an obvious mismatch in alignment and to create the sequence identity matrix. We ran one PERL script (available at https://github.com/zer0liu/bioutils/blob/master/snp/) with alignment as input file to recognize nucleotide variation.

### Phylogenetic Analysis

CDHIT was applied to classify the HAdV genomes in this study into different clusters (Li and Godzik, 2006). The program cd-hit-est was performed with clustering threshold 0.99, n 10, and other parameters set to default. Representative sequences were chosen to run the BLASTN program against the local nucleotide database downloaded from the Genbank database with default parameters. Representative sequences (MW816000 19B0009125) and sequences of the BLASTN program together with several typical HAdV reference strains, 67 genomes in total, were integrated into the dataset for phylogenetic analysis. Dataset alignment was carried out using MAFFT. A maximum-likelihood phylogenetic tree was inferred with RAxML software in CIPRES (Miller et al., 2015) using the GTRGAMMA model with 1,000 bootstraps. The ML tree was displayed in FigTree v1.4.3.

### *In Silico* Restriction Endonuclease Analysis (REA)

*In silico* REA (https://www.bioinformatics.org/sms2/rest_digest.html) was carried out for reference genome (MN307150) and representative genome (MW816000 19B0009125). BamHI, BclI, BglII, EcoRI, HindIII, HpaI, SalI, SmaI, XbaI, and XhoI (Zhao et al., 2014) were chosen for restriction digestion.

## Results

### Epidemiology of HAdV

**Figure 1:**
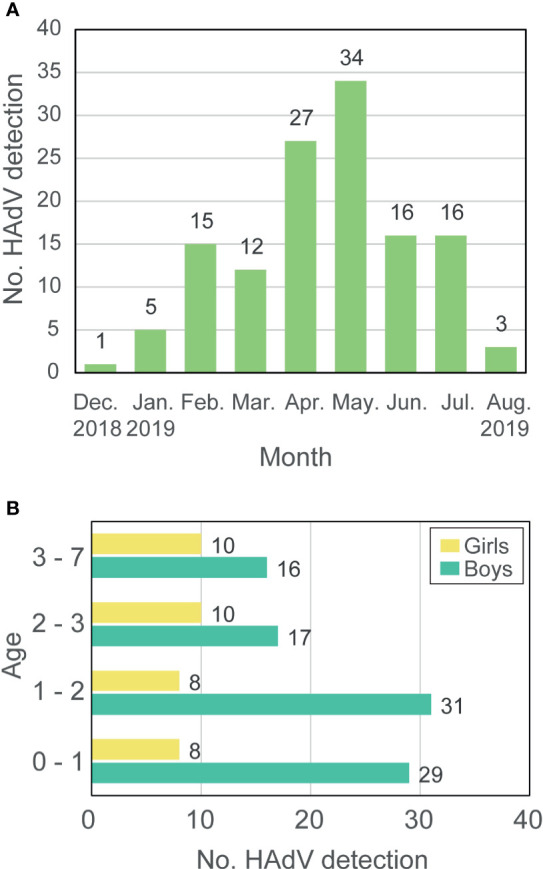
Prevalence of HAdV-7 in Hubei during 2018-2019. **(A)** The number of HAdV detections. **(B)** Age and gender of patients. Labels around the bar represent the number of patients.

HAdV infection was detected in 129 children with severe respiratory symptoms from December 2018 to August 2019 in Wuhan Children’s Hospital, and the number of patients reached a peak in May 2019 (**Figure 1A** and **Supplementary Table 1**). Among all patients, 93 (72.09%) were boys while only 36 (27.91%) were girls with a boy/girl ratio of 2.58:1. The age of patients ranged from three-months-old to seven-years-old with a median age of twenty-months-old. Most patients were younger than 3-years-old and only 26 (20.16%) were older than that. The number of boys that were HAdV-positive was more than that of girls in all age groups (**Figure 1B**). Eight (6.20%) of the examined cases of HAdV infection were fatal in this epidemic, which raises serious concerns due to the catastrophic effect of HAdV infection in children.

### Hemophagocytic Lymphohistiocytosis and Clinical Characteristics of HAdV Infections

**Figure 2:**
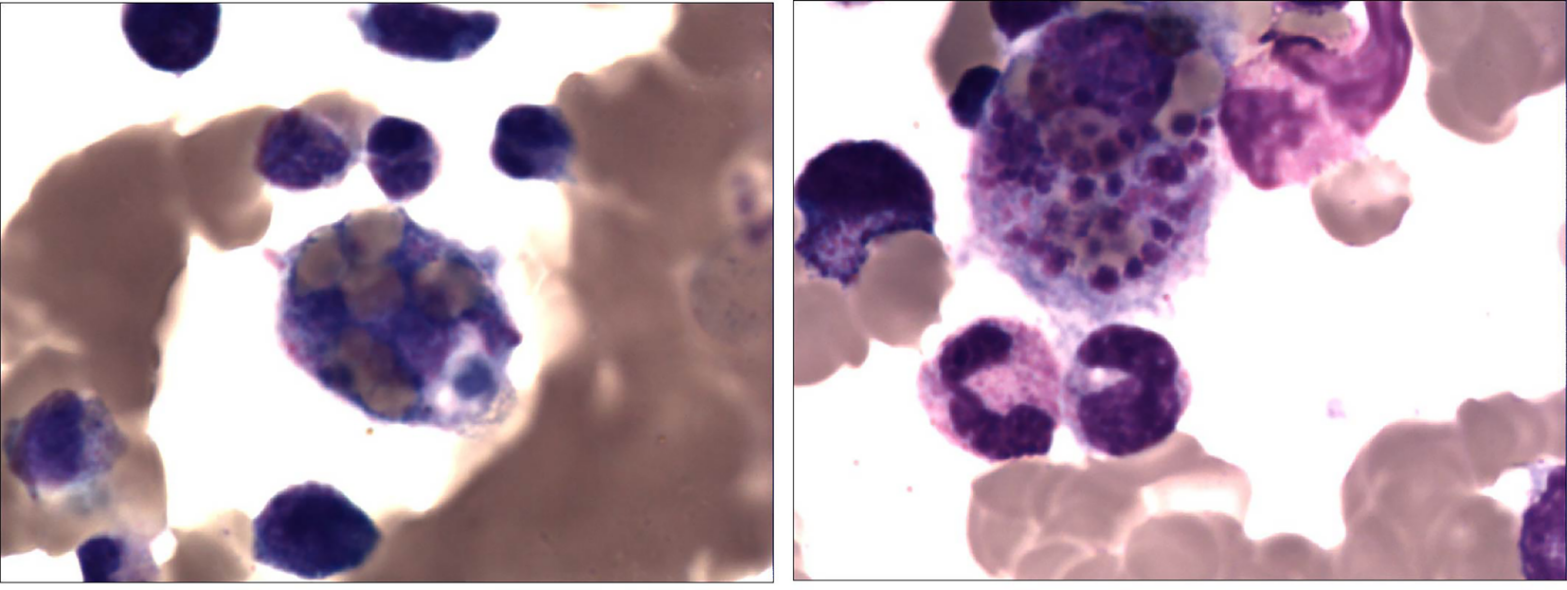
A 2-year-old girl with recurrent fever and cough who was diagnosed with severe adenovirus pneumonia. Bone marrow tissue (×400) showed the marked infiltration of macrophages and hemophagocytosis which suggests hemophagocytic lymphohistiocytosis.

HAdV-related hemophagocytic lymphohistiocytosis was observed in approximately half of the patients (64/129) (**Figure 2** and **Table 1**). To confirm whether HLH was caused by co-infection of other pathogens, a chi-test was carried out to show the independence between HLH and co-infection (**Supplementary Table 2**). There is no significant correlation between them (p-value = 0.18). Based on whether there is HLH or not, all patients were divided into two groups, HLH, and non-HLH, to explore differences in clinical manifestation (**Table 1**) and clinical indicators (**Table 2**).

**Table 1:** Clinical features of HLH and non-HLH patient group.

Clinical features	HLH group n = 64	Non-HLH group n = 65	P-value
Clinical manifestation			
Fever [n (%)]	64 (100.0)	64 (98.4)	–
Peak temperature (> 41°C) [n (%)]	4 (6.2)	2 (3.1)	–
Cough [n (%)]	64 (100.0)	65 (100.0)	–
Wheezing [n (%)]	35 (54.7)	27 (41.5)	0.187
Vomiting [n (%)]	12 (18.8)	8 (12.3)	0.443
Diarrhea [n (%)]	26 (40.6)	18 (27.7)	0.173
Complication			
Myocardial damage [n (%)]	33 (51.6)	20 (30.8)	0.026*
Hepatic dysfunction [n (%)]	26 (40.6)	6 (9.2)	8.71E-05*
Respiratory failure [n (%)]	49 (76.6)	36 (55.4)	0.019*
Cardiac failure [n (%)]	24 (37.5)	7 (10.8)	0.001*
Gastrointestinal bleeding [n (%)]	9 (14.1)	3 (4.6)	–
Toxic encephalopathy [n (%)]	11 (17.2)	6 (9.2)	0.282
ARDS [n (%)]	2 (3.1)	0 (0.0)	–
Acute kidney injury [n (%)]	7 (10.9)	0 (0.0)	–
MODS [n (%)]	4 (6.2)	0 (0.0)	–
Radiographic change			
Consolidation [n (%)]	42 (65.6)	45 (69.2)	0.803
Pleural effusions [n (%)]	22 (34.4)	23 (35.4)	0.949
Treatment			
Oxygen inhalation [n (%)]	44 (68.8)	36 (55.4)	0.167
Continuous positive airway pressure [n (%)]	9 (14.1)	2 (3.1)	–
Mechanical ventilation [n (%)]	8 (12.5)	0 (0.0)	–
Outcome			
Death [n (%)]	7 (10.9)	1 (1.5)	–
ICU admission [n (%)]	40 (62.5)	9 (13.8)	3.56E-08*

ARDS, Acute respiratory distress syndrome; MODS, Multiple organ dysfunction syndrome.

P-value indicates comparisons with significance differences (P ≤ 0.05).

*Significant difference between two groups.

**Table 2:** Clinical characteristics of HLH and non-HLH patient group.

Characteristic	HLH group *n* = 64	Non-HLH group *n* = 65	P-value
Blood routine test			
WBC (× 10^9^/L)	4.37±2.25	6.34±2.46	8.26E-06*
Neutrophils (× 10^9^/L)	104.11±90.15	182.77±124.74	4.00E-04*
Leukocytes (× 10^9^/L)	1.37±0.59	1.84±0.99	4.51E-03*
Platelets (× 10^9^/L)	149.07±70.45	188.89±60.05	1.07E-03*
Hemoglobin (g/L)	92.42±14.8	102.52±11.08	3.12E-05*
Inflammatory indictors			
PCT (ng/ml)	3.16±2.28	2.23±2.14	0.027*
Ferritin (ng/ml)	2470.16±1440.85	936.77±450.3	1.24E-10*
CRP (mg/L)	22.93±17.35	23.65±18.3	0.827
Cytokines			
IL- 2 (pg/ml)	2.1±0.77	1.95±0.55	0.299
IL-4 (pg/ml)	2.89±1.29	2.45±0.92	0.056*
IL-6 (pg/ml)	255.85±224.72	256.0±217.85	0.997
IL-10 (pg/mL)	29.24±15.83	26.19±16.42	0.357
IFN (pg/rnL)	103.39±76.74	108.78±71.42	0.750
TNF-α (pg/ml)	2.2±1.1	2.35±1.1	0.505
Lymphocyte subsets			
CD3 (%)	55.28±11.22	55.2±9.72	0.971
CD4 (%)	29.4±9.7	31.2±8.66	0.494
CD8 (%)	22.42±6.42	21.18±5.98	0.488
CD19 (%)	36.89±12.41	33.75±12.25	0.186
NK (%)	0.06±0.04	0.38±0.96	0.033*
Biochemical testing			
APTI (s)	34.0±9.61	34.97±9.22	0.568
Prothrombin time (PT) (s)	10.74±0.87	10.95±0.85	0.179
Fibrinogen (g/L)	2.36±0.79	2.9±0.79	1.72E-04*
D-dimer (ng/mL)	5.24±3.26	2.82±2.12	1.55E-05*
Triqlyceride (mmol/L)	3.65±1.37	1.8±0.55	9.76E-15*
LDH (U/L)	1511.77±513.59	880.25±344.86	3.01E-12*

WBC, white blood cell; PCT, procalcitonin; CRP, C-reactive protein; IL, interleukin; IFN, interferon; TNF, tumor necrosis factor; APTT, activated partial thromboplastin time; LDH, Lactate dehydrogenase.

P-value indicates comparisons with significance differences (P ≤ 0.05).

*significant difference between two groups.

**Figure 3:**
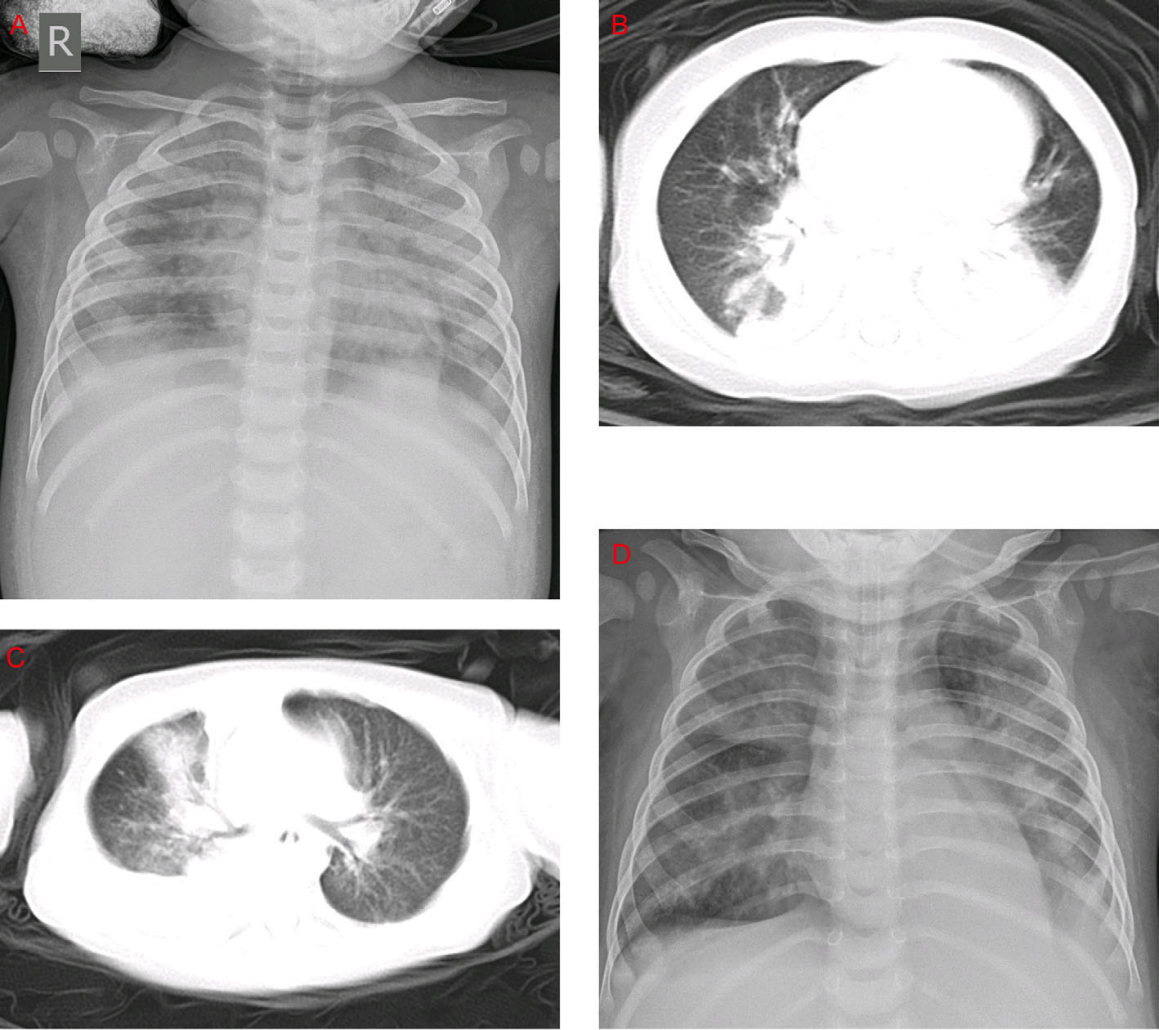
Chest x-rays and chest CTs of patients. **(A)** Chest x-ray of a 1-year-old boy. The brightness of both lungs was diffusely decreased, showing a large area of patchy shadow with uneven density. Tracheal intubation was seen in the trachea and the heart shadow outline was not cl ear. **(B)** Chest CT of a 9-month-old boy: mass shadows of high density in both lungs. Bright bronchogram is seen in the lung tissue area of the lesion, which is also called bronchoinflation sign. Condensation shadows were mainly on the lower right lobe. **(C)** Chest CT of a 11-month-old girl. Condensation shadows were mainly on the right lobe, with right pleural effusion. **(D)** Chest x-ray of a 10-month-old boy. Patchy blurred shadow was seen in both lungs, the left diaphragm and costal phrenic angle disappeared. Tracheal intubation was seen in the trachea and the heart shadow outline was clear.

The most common clinical manifestations of HAdV-7 infections, such as cough, wheezing, vomiting, diarrhea, and fever, including the peak of febrile body temperature (> 41°C), were not significantly different in the two groups. In contrast, among the HLH patients, 76.6% were diagnosed as having respiratory failures, which is significantly higher than that of the non-HLH patients (76.6% *vs* 55.4%, P = 0.019). Several severe outcomes, including respiratory failure, heart failure, and ICU admission were significantly higher in the HLH group compared with the non-HLH group (P < 0.05), but no statistically significant differences were found in toxic encephalopathy. Notably, the rate of mechanical ventilation and death in the HLH group was high. In the chest radiographs, consolidation and pleural effusion were more frequently observed with the non-HLH patients compared to HLH patients, but they did not differ significantly (**Figure 3**).

For blood routine test, patients of the HLH group showed significantly lower levels of leukocytes, platelets, hemoglobin, white blood cells (WBC), and neutrophils (P = 4.51E-03, 1.07E-03, 3.12E-05, 8.26E-06, and 4.00E-04, respectively). For blood inflammatory indicators, lower levels of procalcitonin (PCT) and Ferritin were observed in non-HLH patients than HLH patients (P = 0.027 and 1.24E-10, respectively), while no significant differences were found in C-reactive protein (CRP), interleukin-2, interleukin-4, interleukin-6, interleukin-10, tumor necrosis factor (TNF-α) and interferon (IFN). In terms of cell-mediated immunity, no significant differences were found in CD3, CD4, CD8, CD19 between the two groups of patients, only NK cells were higher in non-HLH patients than HLH patients (P = 0.033). For biochemistry testing, no significant differences were found in prothrombin time (PT) and activated partial thromboplastin time (APPT) between the two groups of patients. However, the level of fibrinogen in the HLH group was lower than that in the non-HLH group (P = 1.72E-04), while the level of D-dimer just displayed the opposite result. We also observed that triglyceride and lactate dehydrogenase had higher values in the HLH group than that in the non-HLH group (P = 9.76E-15 and 3.01E-12, respectively).

### Characteristics of the Viral Genome and Phylogenetic Analysis

To ensure the quality and accuracy of virus genome data, the sequencing depth of each genome was estimated by calculating the average frequency for each base on the genome. HAdV was mapped using MN307150 as the reference genome in all samples. The results showed that the sequencing depth in each sample was greater than 9-fold (**Supplementary Figure S1**). The coverage of the HAdV genome in all samples comes up to at least 99.8%, of which there are 96 samples with full-length coverage of the HAdV genome, suggesting that all HAdV genomes in this study could be used for further analyses.

**Figure 4:**
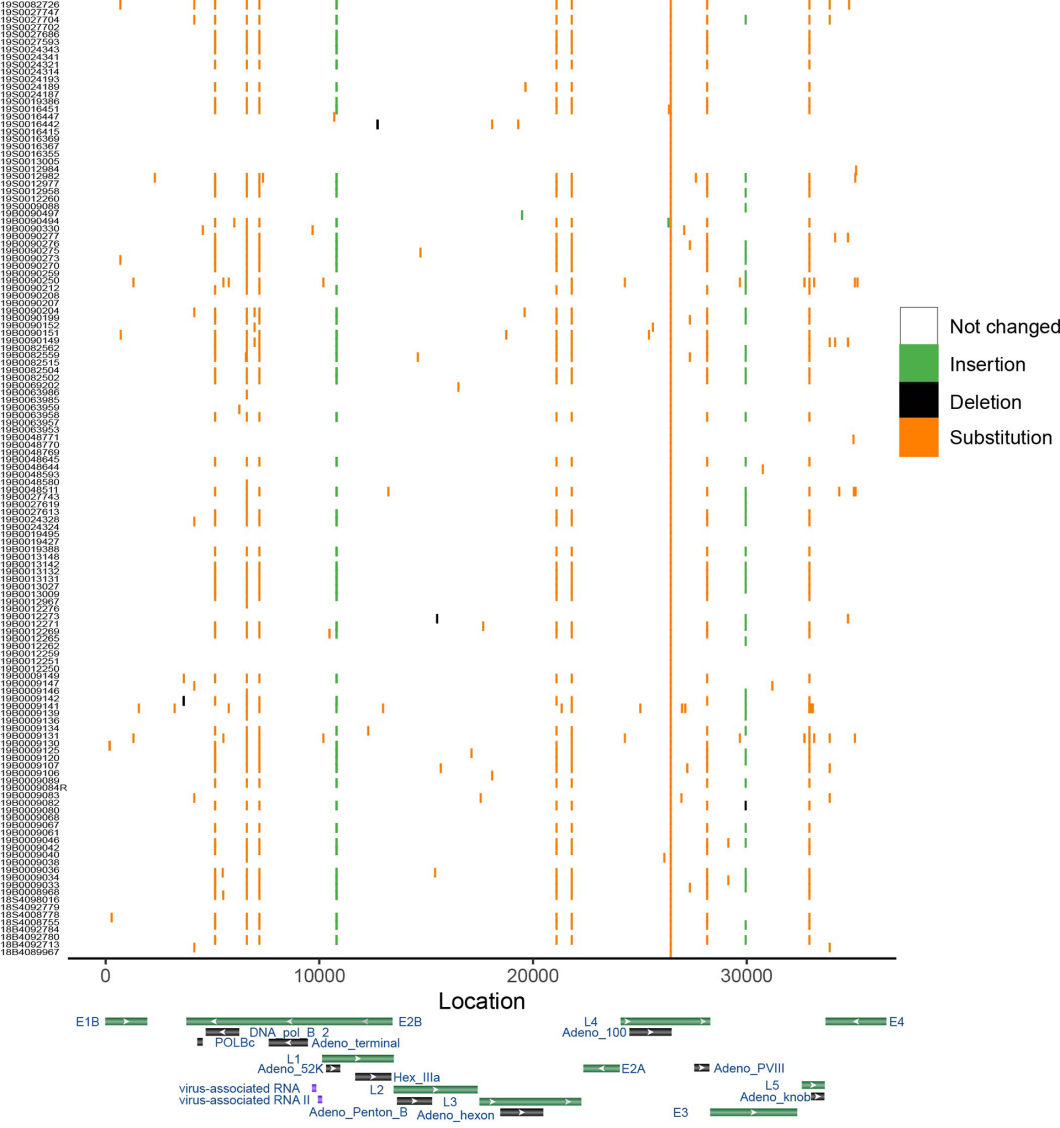
Genome variation of HAdV-7. Genome MN307150 was chosen as the reference. Color shows the type of mutation. X-axis represents the location of genome and y-axis shows the corresponding samples. Gene map displayed several important gene of adenovirus based on the Genbank database.

To show the sequence variations among HAdV-7 subtypes, the results of the point mutation in the genome using MN307150 as the reference genome were displayed (**Figure 4**). The mean number of mutations of other genomes was only 7.37. Substitution is the most frequent type of mutation and deletion was observed only in a few sequences. Some mutations are distributed in a dispersed way in the genome, and it should be noted that there are ten positions in the genome where many mutations were detected in different samples. In the ten positions, seven were transition, two were insertion and one was transversion. The results cannot provide sufficient proof that hemophagocytic syndrome has something to do with specific point mutations.

**Figure 5:**
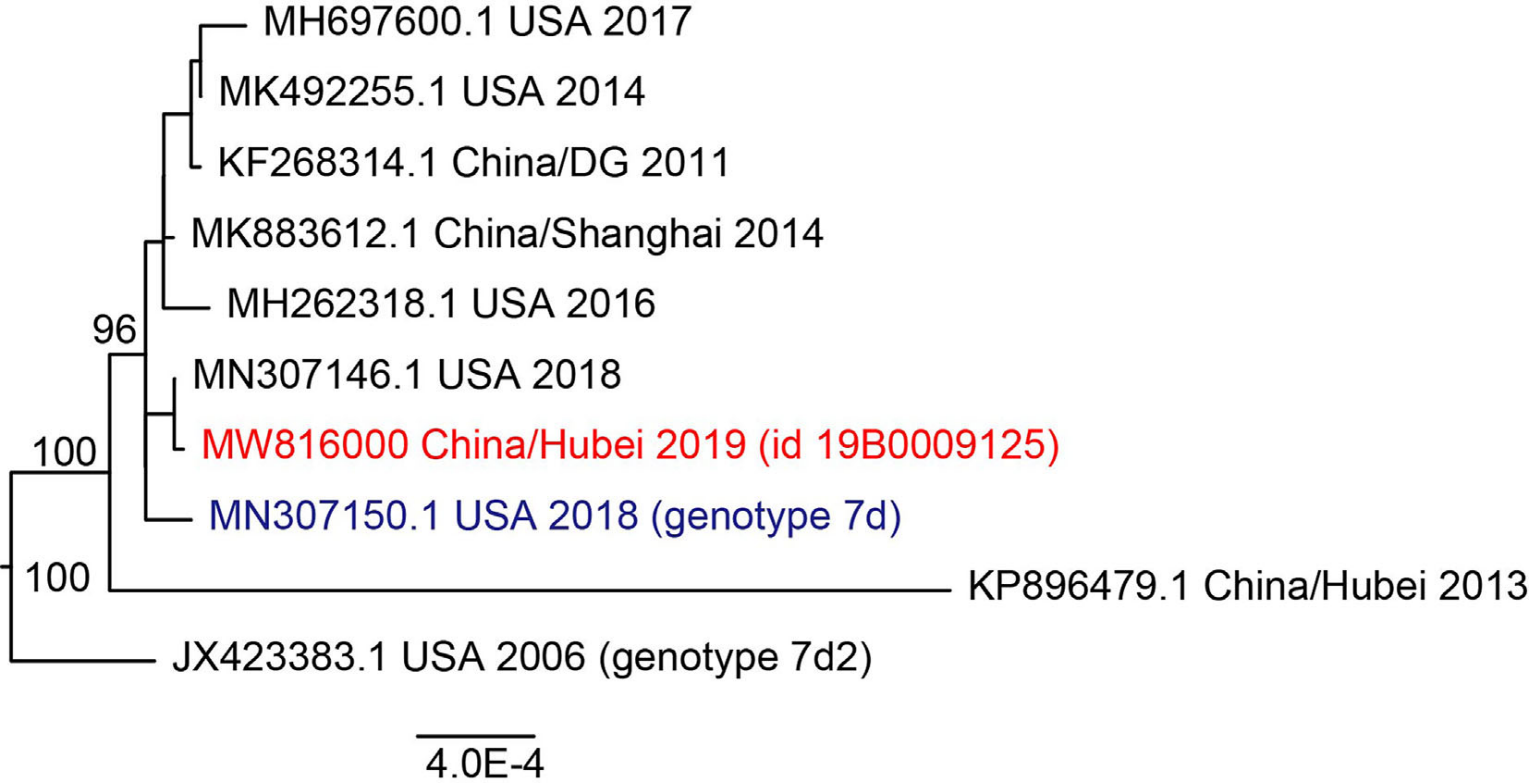
Maximum likelihood phylogenetic tree of whole genome of HAdV 7. Viruses in this study were labeled in red and the reference sequence was labeled in blue. Bootstrap values were shown in tree node.

The minimum nucleotide sequence identity for the whole genome among samples in this study was 99.9%. Considering that HAdV was highly conserved, which might affect phylogenetic analysis, a representative sequence was used in phylogenetic analysis after running the program cd-hit-est. It can be observed that HAdV in this study was clustered with the genome of species B7 and had a close phylogenetic distance with strains isolated in the USA, 2018 rather than strains in China (**Figure 5**). REA showed that the reference genome and representative genome had the same restriction map, suggesting that HAdV in this study belonged to type 7d (**Supplementary Table 3**). We could see that type 7d virus has been detected worldwide in the last thirty years with high genome similarity (**Supplementary Figure 2**), which might be caused by the stability of the double-strand DNA genome in adenovirus. The most recent isolation date of type 7d virus was 2019 in the United States and it was also detected in China in 2018, suggesting that infection by HAdV type 7d might be a global problem.

## Discussion

This study describes a regional outbreak of HAdV-7, of which the genome type was 7d, among children younger than eight years in Hubei Province, China between December 2018 and August 2019. As mentioned in previous studies based on surveillance in China, the highest prevalence of HAdV infections has occurred in the summer months (June to August) (Ouyang et al., 2012; Yao et al., 2019). However, this study observed that the number of HAdV cases detected reached a peak in April and May, suggesting that the epidemic of HAdV has little correlation to season, unlike influenza viruses. HAdV infections are severe in all patients and there are eight fatal cases in this study, confirming that HAdV-7 is a public threat to young people. The mortality rate for HAdV-7 infections varied with time and region. A previous study stated that the mortality rate for children infected by HAdV-7 was up to 18% in China during 1958-1990 and Korea during 1990-2000 but no fatal cases were reported in Chongqing, China during 2009-2014 (Kim et al., 2003; Fu et al., 2019). In this study, the mortality rate is 6.20%, meaning that HAdV is still a threat to the public. An effective vaccine for HAdV-E4 and HAdV-B7 was used by the United States military, which was demonstrated as an effective prevention measure for HAdV infections (Chanock et al., 1966; Russell et al., 2006; Hoke and Snyder, 2013). However, no vaccine is yet supplied for the general public.

HAdV can cause a variety of diseases but hemophagocytic lymphohistiocytosis related to HAdV infections has rarely been documented (Otto et al., 2020; Zhang et al., 2020). In this study, HLH was observed in half of the patients, and the reason for this still needs further investigation. Previous studies have indicated that HAdV-7 is a severe pathogen in children (Lai et al., 2013; Fu et al., 2019). In this study, we found that almost all patients had cough and fever. Moreover, the rate of respiratory failure was very high, especially in patients with HLH. In our study, we found that myocardial damage, followed by hepatic dysfunction, cardiac failure, and toxic encephalopathy, were the most common complications. Remarkably, we also found a higher rate (10.9%) of death associated with HAdV-7 associated HLH.

Previous studies have outlined that HLH might have a connection with cytokine storm and immune imbalance (Odievre et al., 2011; Hosnut et al., 2014; Mellon et al., 2016; Censoplano et al., 2018), but in this study, among all blood inflammatory indicators, only PCT and ferritin displayed higher levels in the HLH group. The criteria for HLH judgment indicate that reduction of whole blood cells, especially the reduction of platelets, is a sign of HLH. The results of this study show that the number of blood cells including leukocytes, platelets, hemoglobin, WBC, and neutrophils in the HLH group were significantly lower than those in the non-HLH group, which was consistent with the criteria. Because the conditions of most patients were severe, it is hard to infer the exact effects of HLH.

As hemophagocytic lymphohistiocytosis is a hyperinflammatory syndrome caused by excessive cytokine release, triggered by genetic or acquired overactivation of macrophages, T and natural killer cells (Canna and Marsh, 2020), we explored the immunity indictor and cytokines in patients with HAdV infection. Regretfully, we found that there was no significant difference between the HLH group and the non-HLH group. We look forward to future studies exploring the reason for HLH occurring secondary to HAdV infection.

We also detected point mutations in the HAdV genome referring to the reference genome. Double-strand DNA genome makes it occur infrequently for point mutation and antigen drift (Mahadevan et al., 2010; Seto et al., 2010) and our results indicate that similarity among all sequences was greater than 99.9% and the mean number of mutations in one genome is 7.37 can also prove this. The insertion of base ‘T’ in the intergenic region was found in around half samples and what this kind of change resulted in for HAdV remained further confirmation. In the L4 gene, a transition was detected in all samples compared with the reference genome, meaning that HAdV in this study might come from the same ancestor. Phylogenetic analysis showed that this group was clustered together with HAdV type 7d because type 7d has become a predominant epidemic strain since 1990 (Li et al., 1996).

HAdV is still a threat to the public, especially to children. Epidemiological studies have reported that HAdV infections account for roughly 5-10% of acute respiratory tract infections in children (Lee et al., 2010b; Jin et al., 2013). Of all adenoviral respiratory infections, HAdV-7 is an epidemic worldwide and occupies about 20% (Erdman et al., 2002; Tang et al., 2011). For public health, we strongly advise that global surveillance of HAdV should be established by whole genome sequencing or other methods. Considering the stability of the HAdV genome and the effective protection of vaccines, it might be important and practicable to vaccinate susceptible populations (Han et al., 2013; Yu et al., 2013). More works, including surveillance, genotyping, genome sequencing, and phylogenetic analysis, need to be carried out to control HAdV outbreaks and protect the global community.

## Data Availability Statement

The datasets presented in this study can be found in online repositories. Genome sequences were deposited in NCBI Genbank database (accession number MW815973 - MW816101). Raw data can be found in National Genomics Data Center (https://ngdc.cncb.ac.cn) with the project ID HRA001060.

## Ethics Statement

The studies involving human participants were reviewed and approved by Medical Ethics Committee of Wuhan Children’s Hospital (Wuhan Maternal and Children Healthcare Hospital). Written informed consent to participate in this study was provided by the participants’ legal guardian/next of kin.

## Author Contributions

All authors collected the clinical data. YL, DW, and JZ drafted the manuscript. XL and DL revised the final manuscript. PH, HD, JX, HeC, YY, and HoC were responsible for collecting samples and clinical data. DW and YY analyzed data. All authors contributed to the article and approved the submitted version.

## Funding

This study was supported by National Key Research and Development Program of China (grant number 2018YFC1603803) and Health Commission of Hubei Province (grant number WJ2021M262).

## Conflict of Interest

The authors declare that the research was conducted in the absence of any commercial or financial relationships that could be construed as a potential conflict of interest.

## Publisher’s Note

All claims expressed in this article are solely those of the authors and do not necessarily represent those of their affiliated organizations, or those of the publisher, the editors and the reviewers. Any product that may be evaluated in this article, or claim that may be made by its manufacturer, is not guaranteed or endorsed by the publisher.
