# Supplementary figures and images for: Human Adenovirus Type 7 Infections in Hubei, China During 2018-2019: Epidemic Features and Genetic Characterization of the Detected Viruses
Source: Front Cell Infect Microbiol. 2021 Aug 19;11:684606. doi: 10.3389/fcimb.2021.684606 (PMC8417316; doi:10.3389/fcimb.2021.684606)

Sample ID

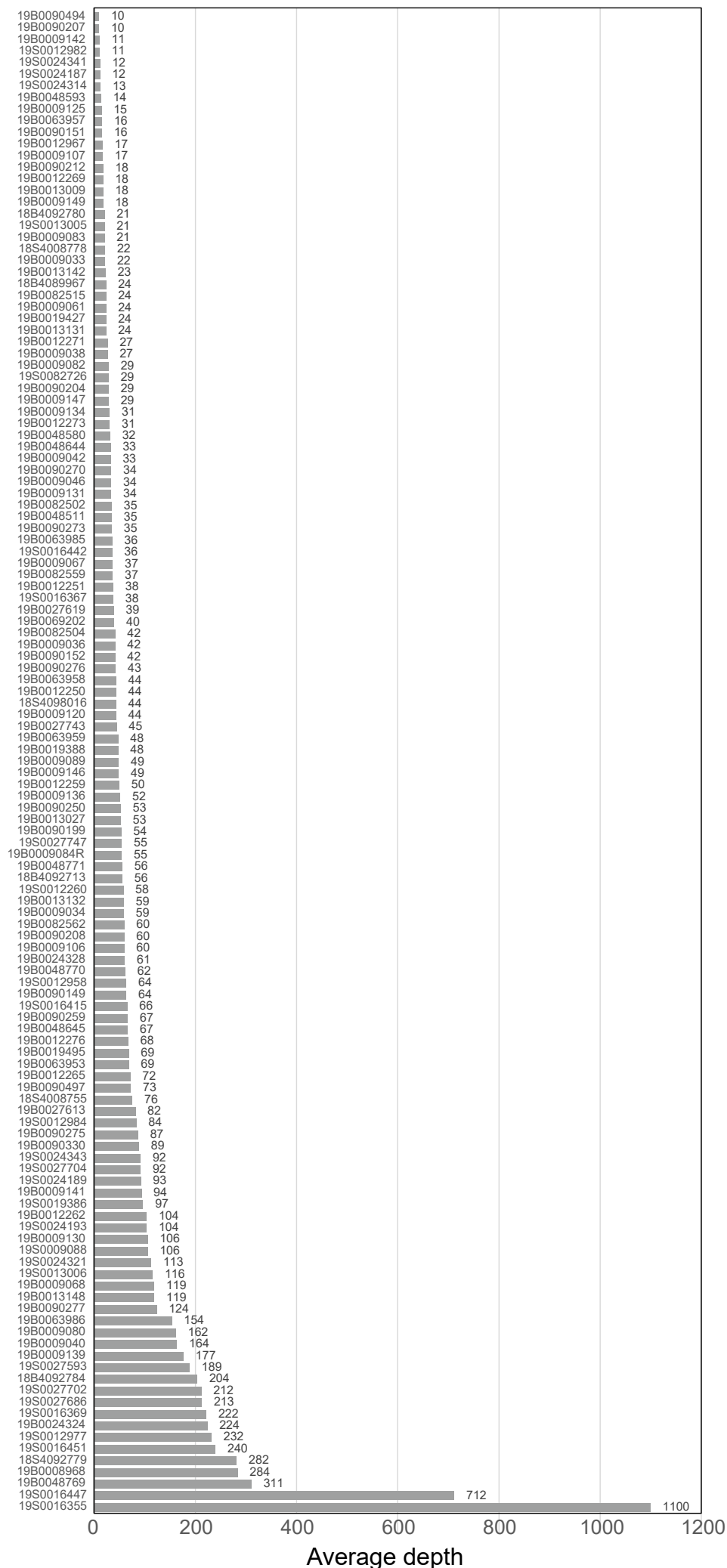

Supplement: Supplementary file 1 [file DataSheet_1.pdf]

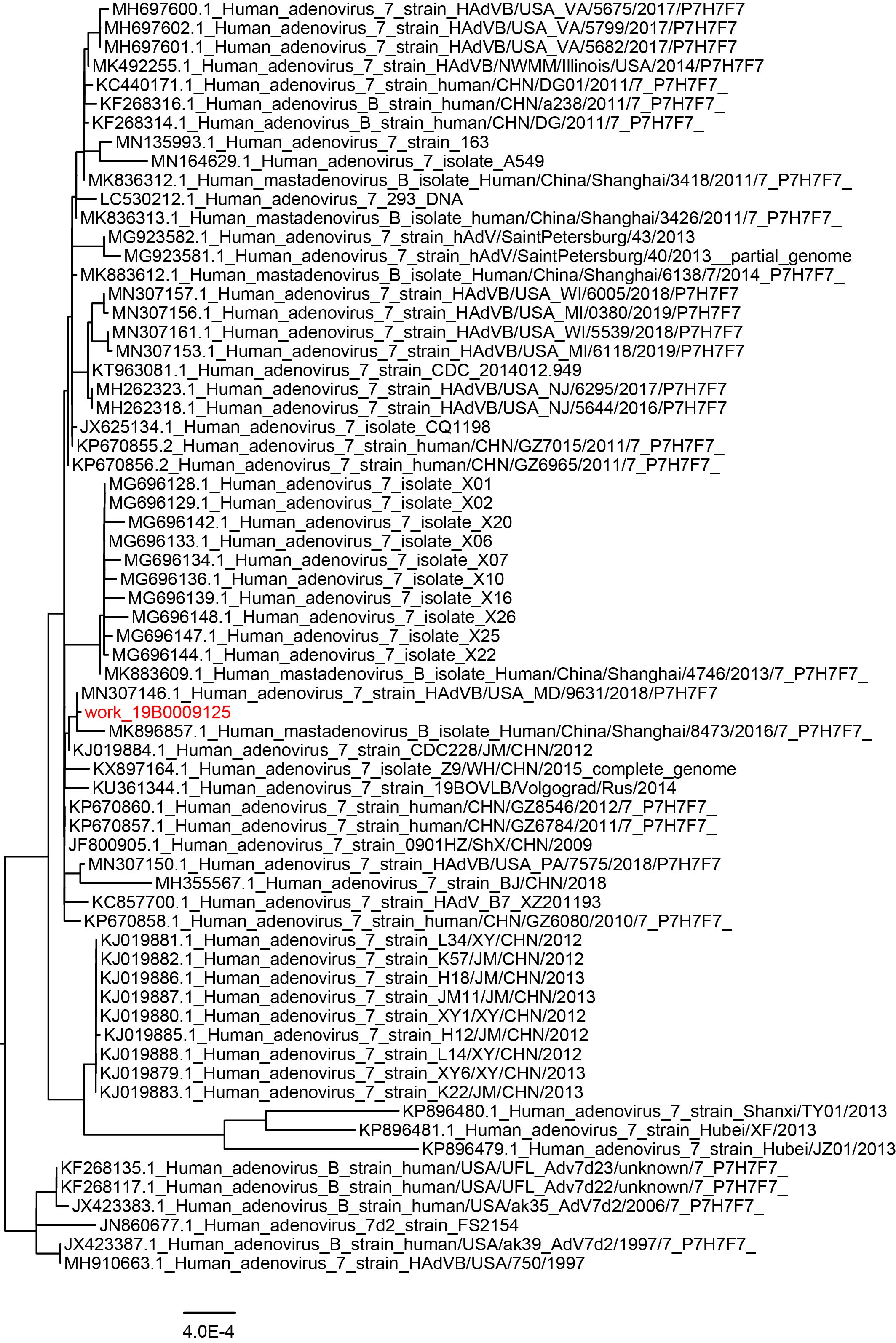

Supplement: Supplementary file 3 [file Image_1.jpeg]
