# Supplementary material for: Human Adenovirus Type 7 Infections in Hubei, China During 2018-2019: Epidemic Features and Genetic Characterization of the Detected Viruses
Source: Front Cell Infect Microbiol. 2021 Aug 19;11:684606. doi: 10.3389/fcimb.2021.684606 (PMC8417316; doi:10.3389/fcimb.2021.684606)
Supplement: Supplementary file 2 [file DataSheet_2.docx]

**Supplementary Table 1. Detail information of samples**

| Accession number | Sample ID | Raw data filename | Gender | Age | Admission date | Hemophagocytic lymphohistiocytosis | Co-infection | Comments |
| --- | --- | --- | --- | --- | --- | --- | --- | --- |
| MW815979 | 18S4092779 | N100006704_L01_41 | Girl | 7 y 9 m | 2019-01-10 | Y | Mycoplasma | - |
| MW815974 | 18B4092713 | N100006690_L01_29 | Boy | 9 m 0 d | 2019-01-15 | Y | - | - |
| MW816020 | 19B0012967 | V300020472_L04_19 | Boy | 9 m 22 d | 2019-01-19 | Y | Hemophilus influenza | Death |
| MW815980 | 18S4098016 | N100010594_L01_5 | Boy | 9 m 11 d | 2019-01-28 | N | - | - |
| MW816081 | 19S0016355 | V300013940_L03_38 | Boy | 10 m 4 d | 2019-01-29 | Y | Candida | - |
| MW816084 | 19S0016415 | N100007764_L01_45 | Girl | 9 m 30 d | 2019-02-01 | Y | - | - |
| MW815975 | 18B4092780 | V300013984_L03_44 | Boy | 5 y 2 m | 2019-02-02 | Y | - | - |
| MW815976 | 18B4092784 | V300014140_L04_21 | Boy | 8 m 21 d | 2019-02-06 | Y | - | - |
| MW816085 | 19S0016442 | N100006722_L01_25 | Girl | 2 y 5 m | 2019-02-10 | Y | Hemophilus influenza, Rotaviral enteritis, Candida | - |
| MW816087 | 19S0016451 | V300013992_L03_31 | Girl | 4 y 2 m | 2019-02-11 | N | Hemophilus influenza | - |
| MW815977 | 18S4008755 | V300013896_L02_21 | Boy | 10 m 12 d | 2019-02-12 | Y | - | - |
| MW816082 | 19S0016367 | V300015743_L03_38 | Girl | 11 m 30 d | 2019-02-12 | Y | Mycoplasma | - |
| MW816076 | 19S0012977 | N100008024_L01_22 | Boy | 1 y 6 m | 2019-02-13 | Y | - | Death |
| MW816078 | 19S0012984 | V300016028_L01_17 | Girl | 1 y 6 m | 2019-02-15 | Y | Fungi | - |
| MW816086 | 19S0016447 | V300014118_L02_33 | Boy | 1 y 7 m | 2019-02-17 | Y | Flu | - |
| MW816079 | 19S0013005 | V300009624_L01_19 | Girl | 2 y 1 m | 2019-02-20 | N | Mycoplasma, Hemophilus influenza | - |
| MW816083 | 19S0016369 | N100008097_L01_1 | Girl | 1 y 4 m | 2019-02-20 | N | Mycoplasma, Hemophilus influenza | - |
| MW816089 | 19S0024187 | N100008097_L01_3 | Girl | 1 y 9 m | 2019-02-20 | N | Mycoplasma | - |
| MW816090 | 19S0024189 | N100007744_L01_9 | Boy | 1 y 2 m | 2019-02-21 | Y | Candida | - |
| MW816077 | 19S0012982 | V300016026_L04_29 | Boy | 2 y 5 m | 2019-02-27 | N | - | - |
| MW816080 | 19S0013006 | V300016107_L03_37 | Boy | 1 y 2 m | 2019-03-03 | Y | - | - |
| MW816091 | 19S0024193 | V300007961_L03_1 | Girl | 2 y 4 m | 2019-03-03 | N | Mycoplasma | - |
| MW816021 | 19B0013009 | V300016074_L01_17 | Boy | 8 m 3 d | 2019-03-06 | N | Respiratory syncytial virus | - |
| MW816022 | 19B0013027 | V300016097_L01_8 | Girl | 2 y | 2019-03-07 | Y | Candida, Mycoplasma | - |
| MW816092 | 19S0024314 | V300016072_L04_32 | Boy | 3 y 4 m | 2019-03-12 | N | Mycoplasma | - |
| MW816093 | 19S0024321 | V300015745_L01_6 | Boy | 3 y 3 m | 2019-03-19 | N | - | - |
| MW816030 | 19B0024324 | V300009624_L03_1 | Boy | 3 y 4 m | 2019-03-22 | N | - | - |
| MW816031 | 19B0024328 | N100008335_L01_16 | Boy | 1 y 1 m | 2019-03-23 | Y | - | - |
| MW816095 | 19S0024343 | V300015534_L02_18 | Boy | 2 y 8 m | 2019-03-25 | N | Mycoplasma | - |
| MW816094 | 19S0024341 | V300015534_L02_22 | Boy | 3 y 1 m | 2019-03-26 | N | - | - |
| MW816075 | 19S0012958 | V300015796_L01_33 | Girl | 1 y 9 m | 2019-03-27 | Y | Fungi | - |
| MW816019 | 19B0012276 | V300015102_L04_23 | Boy | 1 y 1 m | 2019-03-29 | Y | Mycoplasma, | - |
| MW816032 | 19B0027613 | V300015106_L04_47 | Boy | 2 y 3 m | 2019-04-02 | Y | Mycoplasma | - |
| MW816033 | 19B0027619 | V300015806_L03_7 | Boy | 8 m 16 d | 2019-04-07 | Y | - | - |
| MW816024 | 19B0013132 | V300016150_L03_8 | Girl | 2 y 9 m | 2019-04-08 | N | Mycoplasma | - |
| MW816088 | 19S0019386 | V300015388_L03_14 | Boy | 1 y 4 m | 2019-04-09 | Y | - | - |
| MW815983 | 19B0009034 | V300016150_L03_5 | Boy | 7 m 21 d | 2019-04-11 | Y | Mycoplasma | - |
| MW816023 | 19B0013131 | N100008891_L01_33 | Boy | 2 y 3 m | 2019-04-13 | Y | - | - |
| MW816027 | 19B0019388 | V300015388_L04_6 | Boy | 1 y 10 m | 2019-04-15 | N | - | - |
| MW815984 | 19B0009036 | V300015388_L03_17 | Boy | 1 y 1 m | 2019-04-16 | N | - | - |
| MW815985 | 19B0009038 | V300018553_L02_23 | Boy | 3 y 7 m | 2019-04-16 | Y | Mycoplasma, | - |
| MW816035 | 19B0048511 | V300016022_L04_7 | Girl | 3 y 11 m | 2019-04-16 | Y | Mycoplasma | - |
| MW816018 | 19B0012273 | V300016001_L03_33 | Boy | 3 m 9 d | 2019-04-17 | N | Cytomegalovirus | - |
| MW816096 | 19S0027593 | V300015249_L04_15 | Boy | 4 m 27 d | 2019-04-18 | Y | Fungi | Death |
| MW815986 | 19B0009040 | V300018553_L04_25 | Girl | 10 m 1 d | 2019-04-19 | Y | Cytomegalovirus | - |
| MW815982 | 19B0009033 | N100008684_L01_43 | Girl | 2 y 10 m | 2019-04-20 | N | - | - |
| MW816025 | 19B0013142 | V300016001_L03_40 | Boy | 10 m 9 d | 2019-04-20 | N | - | - |
| MW816100 | 19S0027747 | V300016103_L04_29 | Boy | 1 y 4 m | 2019-04-20 | N | - | - |
| MW815988 | 19B0009046 | V300018553_L04_33 | Boy | 6 m 27 d | 2019-04-22 | Y | Hemophilus influenza | - |
| MW816034 | 19B0027743 | V300016022_L04_8 | Boy | 1 y 9 m | 2019-04-22 | N | - | - |
| MW816098 | 19S0027702 | V300015813_L03_41 | Boy | 1 y 5 m | 2019-04-23 | Y | - | - |
| MW815987 | 19B0009042 | V300018553_L04_24 | Boy | 2 y 5 m | 2019-04-25 | Y | Candida | - |
| MW816028 | 19B0019427 | V300015997_L04_32 | Girl | 2 y 5 m | 2019-04-25 | N | Fungi | - |
| MW816099 | 19S0027704 | N100008448_L01_17 | Boy | 9 m 9 d | 2019-04-25 | Y | - | Death |
| MW816012 | 19B0012251 | V300015997_L04_33 | Boy | 2 y 5 m | 2019-04-26 | Y | Mycoplasma, Epstein-Barr virus, | - |
| MW816097 | 19S0027686 | V300015989_L02_46 | Boy | 10 m 7 d | 2019-04-26 | Y | Fungi | Death |
| MW815990 | 19B0009067 | V300020505_L04_5 | Boy | 1 y 2 m | 2019-04-29 | Y | Candida | - |
| MW816010 | 19B0009149 | V300015997_L04_29 | Boy | 2 y 7 m | 2019-04-29 | N | - | - |
| MW816007 | 19B0009142 | V300020482_L02_31 | Boy | 9 m 29 d | 2019-04-30 | N | - | - |
| MW816005 | 19B0009139 | V300020482_L02_33 | Girl | 2 y 8 m | 2019-05-01 | N | Candida | - |
| MW816006 | 19B0009141 | V300020482_L02_30 | Boy | 1 y 6 m | 2019-05-01 | N | Mycoplasma | - |
| MW815981 | 19B0008968 | V300020312_L01_4 | Girl | 6 m 14 d | 2019-05-03 | Y | - | - |
| MW815991 | 19B0009068 | V300020505_L04_2 | Boy | 1 y 3 m | 2019-05-03 | N | - | - |
| MW816074 | 19S0012260 | V300020498_L03_32 | Boy | 6 m 22 d | 2019-05-06 | Y | Fungi, | - |
| MW816029 | 19B0019495 | V300020303_L04_22 | Girl | 10 m 1 d | 2019-05-07 | N | - | - |
| MW815989 | 19B0009061 | V300020376_L03_36 | Boy | 1 y 2 m | 2019-05-09 | N | - | - |
| MW816011 | 19B0012250 | V300020315_L04_15 | Boy | 3 y 8 m | 2019-05-09 | Y | Mycoplasma | - |
| MW816009 | 19B0009147 | N100008731_L01_18 | Girl | 5 y 9 m | 2019-05-11 | Y | Aspergillus | - |
| MW816036 | 19B0048580 | V300020305_L03_27 | Girl | 5 m 18 d | 2019-05-12 | N | - | - |
| MW816008 | 19B0009146 | V300020347_L01_19 | Boy | 1 y 1 m | 2019-05-13 | N | - | - |
| MW816017 | 19B0012271 | V300020373_L01_13 | Boy | 2 y 6 m | 2019-05-13 | N | Hemophilus influenza | - |
| MW815992 | 19B0009080 | V300020307_L02_32 | Boy | 3 y 9 m | 2019-05-14 | Y | Mycoplasma | - |
| MW815997 | 19B0009106 | N100008009_L01_25 | Boy | 1 y 3 m | 2019-05-14 | N | - | - |
| MW815998 | 19B0009107 | N100009093_L01_25 | Boy | 11 m 4 d | 2019-05-14 | N | Mycoplasma | - |
| MW815999 | 19B0009120 | N100009039_L01_42 | Boy | 2 y 1 m | 2019-05-15 | Y | - | - |
| MW816001 | 19B0009130 | V300020378_L03_33 | Girl | 3 y 10 m | 2019-05-15 | N | Mycoplasma, Hemophilus influenza | - |
| MW816037 | 19B0048593 | V300020307_L01_35 | Girl | 1 y 7 m | 2019-05-15 | N | - | - |
| MW816038 | 19B0048644 | V300020378_L03_32 | Boy | 1 y 8 m | 2019-05-17 | N | Mycoplasma, Epstein-Barr virus | - |
| MW816039 | 19B0048645 | V300020378_L03_31 | Boy | 2 y 6 m | 2019-05-17 | N | Mycoplasma, Epstein-Barr virus, Parainfluenza virus | - |
| MW816073 | 19S0009088 | N100010207_L01_16 | Boy | 4 y 6 m | 2019-05-17 | Y | Mycoplasma | Death |
| MW815993 | 19B0009082 | V300020278_L01_1 | Boy | 1 y 11 m | 2019-05-18 | N | - | - |
| MW816002 | 19B0009131 | N100008870_L01_22 | Boy | 10 m 13 d | 2019-05-18 | Y | - | - |
| MW815995 | 19B0009084R | N100009072_L01_23 | Boy | 5 y 9 m | 2019-05-19 | N | Epstein-Barr virus | - |
| MW815994 | 19B0009083 | N100009140_L01_22 | Boy | 1 y 8 m | 2019-05-20 | N | - | - |
| MW816016 | 19B0012269 | V300020365_L04_12 | Girl | 3 y 5 m | 2019-05-22 | Y | Epstein-Barr virus | - |
| MW816014 | 19B0012262 | V300020279_L04_4 | Boy | 1 y 8 m | 2019-05-23 | Y | - | - |
| MW816015 | 19B0012265 | V300020365_L02_17 | Girl | 2 y 8 m | 2019-05-23 | Y | - | - |
| MW816004 | 19B0009136 | V300020309_L03_34 | Boy | 9 m 11 d | 2019-05-25 | N | - | - |
| MW816000 | 19B0009125 | V300020262_L01_9 | Boy | 4 y 5 m | 2019-05-26 | N | Mycoplasma | - |
| MW816003 | 19B0009134 | V300020309_L03_35 | Boy | 1 y 10 m | 2019-05-26 | Y | Acinetobacter baumannii | - |
| MW816013 | 19B0012259 | N100010496_L01_38 | Boy | 1 y 4 m | 2019-05-26 | Y | - | - |
| MW816050 | 19B0082502 | V300020481_L01_12 | Boy | 6 m 23 d | 2019-05-29 | Y | Cytomegalovirus, Epstein-Barr virus, Mycoplasma | - |
| MW816052 | 19B0082515 | V300020488_L03_40 | Boy | 8 m | 2019-05-31 | Y | Parainfluenza virus, Hemophilus influenza, Aspergillus, | - |
| MW816051 | 19B0082504 | V300020256_L03_21 | Boy | 2 y 5 m | 2019-06-01 | N | Epstein-Barr virus | - |
| MW816042 | 19B0048771 | V300020314_L03_2 | Boy | 2 y 3 m | 2019-06-03 | N | - | - |
| MW816041 | 19B0048770 | V300020314_L02_6 | Boy | 1 y 6 m | 2019-06-04 | N | - | - |
| MW816040 | 19B0048769 | V300020314_L04_12 | Girl | 3 y 8 m | 2019-06-05 | Y | - | - |
| MW816101 | 19S0082726 | N100010261_L01_5 | Boy | 4 y | 2019-06-07 | N | - | - |
| MW816026 | 19B0013148 | V300020331_L03_24 | Boy | 2 y 5 m | 2019-06-13 | Y | Mycoplasma, Epstein-Barr virus, Parvovirus B19 | - |
| MW815996 | 19B0009089 | V300020476_L04_33 | Boy | 2 y 8 m | 2019-06-14 | Y | Mycoplasma | - |
| MW815973 | 18B4089967 | V300021373_L04_9 | Boy | 7 m 20 d | 2019-06-21 | Y | - | - |
| MW816043 | 19B0063953 | N100010260_L01_44 | Boy | 1 y 3 m | 2019-06-22 | N | - | - |
| MW816053 | 19B0082559 | N100010342_L01_22 | Girl | 2 y 6 m | 2019-06-22 | N | Candida | - |
| MW816054 | 19B0082562 | V300021198_L04_6 | Boy | 6 y 8 m | 2019-06-24 | N | Mycoplasma | - |
| MW816047 | 19B0063985 | V300021332_L03_38 | Boy | 1 y 8 m | 2019-06-26 | Y | - | - |
| MW816048 | 19B0063986 | V300021332_L04_37 | Boy | 11 m 13 d | 2019-06-26 | Y | Aspergillus fumigatus | Death |
| MW816056 | 19B0090151 | N100010362_L01_19 | Boy | 6 m 6 d | 2019-06-28 | N | - | - |
| MW816055 | 19B0090149 | N100010376_L01_17 | Boy | 2 y 10 m | 2019-06-29 | Y | Mycoplasma | - |
| MW816057 | 19B0090152 | N100010362_L01_17 | Girl | 10 m 9 d | 2019-06-29 | Y | - | - |
| MW816046 | 19B0063959 | V300024366_L03_35 | Boy | 1 y 9 m | 2019-07-01 | N | Fungi, Parainfluenza virus | - |
| MW816044 | 19B0063957 | V300024366_L04_39 | Boy | 4 y 4 m | 2019-07-03 | N | - | - |
| MW816045 | 19B0063958 | V300024366_L04_40 | Girl | 5 y 6 m | 2019-07-03 | N | Mycoplasma | - |
| MW816058 | 19B0090199 | V300021369_L04_40 | Boy | 6 m 6 d | 2019-07-04 | Y | Candida | - |
| MW816063 | 19B0090250 | V300024241_L03_46 | Boy | 1 y 3 m | 2019-07-04 | Y | Fungi, Candida | - |
| MW816060 | 19B0090207 | N100010286_L01_19 | Boy | 11 m 26 d | 2019-07-05 | N | Mycoplasma | - |
| MW816059 | 19B0090204 | N100010195_L01_17 | Girl | 4 y 4 m | 2019-07-06 | N | Mycoplasma | - |
| MW816069 | 19B0090277 | V300024241_L02_39 | Boy | 7 y 8 m | 2019-07-07 | N | Mycoplasma | - |
| MW816061 | 19B0090208 | N100010455_L01_18 | Boy | 6 y 10 m | 2019-07-09 | N | Mycoplasma | - |
| MW816062 | 19B0090212 | V300023641_L04_43 | Boy | 2 y 10 m | 2019-07-12 | N | - | - |
| MW816067 | 19B0090275 | V300021289_L03_33 | Boy | 1 y 2 m | 2019-07-15 | Y | Parainfluenza virus | - |
| MW816070 | 19B0090330 | V300023783_L04_12 | Boy | 1 y 5 m | 2019-07-15 | Y | Fungi | - |
| MW816068 | 19B0090276 | V300021289_L03_34 | Girl | 1 y 9 m | 2019-07-18 | Y | - | - |
| MW816066 | 19B0090273 | N100010693_L01_26 | Boy | 9 m 3 d | 2019-07-20 | N | - | - |
| MW816065 | 19B0090270 | V300024386_L04_42 | Girl | 6 m 3 d | 2019-07-21 | N | - | - |
| MW816064 | 19B0090259 | V300025722_L04_40 | Boy | 9 m 9 d | 2019-07-29 | N | - | - |
| MW816072 | 19B0090497 | V300026491_L04_8 | Girl | 1 y 7 m | 2019-08-14 | Y | Mycoplasma | Death |
| MW816049 | 19B0069202 | V300026456_L01_3 | Girl | 1 y 1 m | 2019-08-18 | N | Mycoplasma | - |
| MW816071 | 19B0090494 | V300026622_L04_5 | Boy | 2 y 7 m | 2019-08-26 | N | Mycoplasma | - |
| MW815978 | 18S4008778 | V300007966_L01_6 | Girl | 4 y | 2018-12-26 | N | Mycoplasma | - |

**Supplementary Table 2. Chi-test between HLH and co-infection**

| Co-infection | Y | N |
| --- | --- | --- |
| HLH |  |  |
| Y | 40 | 24 |
| N | 32 | 33 |

chi-square = 1.796

p = 0.180

**Supplementary Table 3. The in-silico restriction endonuclease analysis (REA) pattern between reference genome and representative genome**

|  | MN307150.1 | 19B0009125 |
| --- | --- | --- |
| BamHI | 13139, 8115, 4349, 3460, 2598, 1164, 947, 737, 716 | 13144, 8116, 4349, 3460, 2598, 1164, 947, 737, 716 |
| BclI | 11521, 6794, 5891, 3188, 2836, 2729, 1192, 1074 | 11526, 6794, 5891, 3188, 2836, 2730, 1192, 1074 |
| BglII | 7404, 5302, 3577, 3091, 2624, 2386, 2333, 2200, 1846, 1555, 1280, 1041, 586 | 7404, 5303, 3577, 3091, 2624, 2386, 2333, 2200, 1851, 1555, 1280, 1041, 586 |
| EcoRI | 30489, 4736 | 30495, 4736 |
| HindIII | 7078, 5697, 4711, 4486, 3422, 3206, 2173, 1710, 1375, 1329, 38 | 7083, 5698, 4711, 4486, 3422, 3206, 2173, 1710, 1375, 1329, 38 |
| HpaI | 24425, 7234, 3566 | 24430, 7235, 3566 |
| SalI | 17323, 11555, 6347 | 17328, 11556, 6347 |
| SmaI | 12154, 6766, 4608, 3675, 2483, 2413, 2088, 1038 | 12159, 6767, 4608, 3675, 2483, 2413, 2088, 1038 |
| XbaI | 10381, 8859, 7094, 4552, 4339 | 10381, 8859, 7095, 4557, 4339 |
| XhoI | 18377, 8288, 3789, 2526, 1222, 623, 400 | 18382, 8289, 3789, 2526, 1222, 623, 400 |
